# Case Study: Using a Shared International Database to Document Veterinary Consumption of Antibiotics in Pakistan

**DOI:** 10.3390/antibiotics12020394

**Published:** 2023-02-15

**Authors:** Mashkoor Mohsin, Umar Farooq, Maria Hartmann, Sandra Brogden, Lothar Kreienbrock, Julia Stoffregen

**Affiliations:** 1 Institute of Microbiology, University of Agriculture, Faisalabad 38000, Pakistan; 2 NS Poultry, Faisalabad 38000, Pakistan; 3 Department of Biometry, Epidemiology and Information Processing, University of Veterinary Medicine Hannover, 30559 Hannover, Germany

**Keywords:** AMU, shared database, AMR, VetCAb-ID, Pakistan

## Abstract

In this paper, we present a case study of Pakistan documenting the use of antimicrobial drugs in poultry flocks in the VetCAb-ID database. Unlike other databases, this system allows international users to upload their data directly. Based on expert interviews and a review of the latest publications on the topic, we provide an alternative approach to harmonizing data collection among countries. This paper will provide impetus to formulate joint requirement documentation for an AMU database on a global level that international users can adapt for their own purposes and projects.

## 1. Introduction

In recent years, Pakistan has upsurged its ambitions to combat the antimicrobial resistance (AMR) of bacterial pathogens. Monitoring antimicrobial drug usage (AMU) at the farm level is the first and indispensable step to achieving this goal. This case study presents how the international VetCAb-ID (Veterinary Consumption of Antibiotics—International Documentation) database was used and adapted for the digital monitoring of AMU in Pakistan. Monitoring AMU in the database presented here involves the systematic documentation of data such as frequency, amount and active components of antibiotics, animal species treated, periods of harvesting and other additional data points. Bringing data together facilitates the analysis of metrics such as treatment incidence and daily dose of drugs used, providing benchmarking measures for farms, regions and countries with regard to AMU [1].

Part of this study focuses on organizing the direct data input of VetCAb-ID users and thus follows a recent shift in perspective within the community. While analyzing AMU/AMR was used to develop corresponding policies for collective action, discussions are now moving towards elaborating how to generate a common digital infrastructure to collect data on a global scale (e.g., the European Surveillance of Veterinary Antimicrobial Consumption (ESVAC) or the World Organisation for Animal Health (WOAH) global databases [2,3,4,5]). One such database to mention in this respect is TISSA (Tripartite Integrated System for Surveillance on AMR and AMU), organized by the Alliance for One Health of the World Health Organisation (WHO) together with the WOAH, the Food and Agriculture Organization of the United Nations (FAO) and the United Nations Environment Programme (UNEP). TISSA aims to provide user-friendly access to a harmonized collection and analysis of AMU and AMR on a global and regional scale [6,7]. As might be expected, there will be several challenges related to the standardization and harmonization processes behind such a database. In this respect, sharing experiences in using such databases is critical for the continuous development and optimization of such systems. However, surprisingly, there are only a few studies from the perspective of users that elaborate on the feasibility of database programs used for documenting the use of antimicrobial drugs [1,8]. Since numerous problems concerning sharing secondary data are known [1,3,9], one would expect that using a database for harmonized documentation of primary data on a global level would have its own intricacies.

This study elaborates on this assumption and aims to share experiences of using the international VetCAb-ID database in Pakistan. For example, challenges, benefits, sketches from the database, user roles and examples of data from two farms in Pakistan are provided and discussed. To compare and contrast the findings of this case study with the results of previous case studies, a literature review was conducted. Here, we have found that VetCAb-ID is one of the few databases that allows users to collect primary data through direct entry. Hence, the challenges and solutions presented may be seen and discussed as an alternative data collection approach to consider for future shared global databases.

## 2. Results

The results of this study begin with findings from the literature review, which describe the types of shared databases and related challenges and benefits that have already been discussed in the literature. Subsequently, results from our case study, including the expert interview and descriptive analysis of the poultry data of Pakistan in VetCAb-ID, are provided.

### 2.1. Results from the Literature Review

In general, publications evaluate databases for AMU/AMR at the farm [1] or national level [2,3], as well as within one [2,3] or among several European countries [1,3,5,8]. While databases for international use are mentioned (e.g., ESVAC, [1,6]), it is not further evaluated how the data are provided to the system. For ESVAC, for example, the transfer of data is voluntary and stems from national or regional data collection systems. Hence, users need to compile the data into another database first [5]. Sanders et al. [1] and Mesa Varona et al. [3] show how these data collection systems may vary among regions. For example, differences include the scope of registered animals, the method of documenting treatments or daily doses, among others [1,3].

A feasibility study of a data collection system in Germany was published at the national level. The system was considered adequate when the data allowed for comparisons among specific entities, the calculation of descriptive statistics and the analysis of how to generalize upon the results [2]. However, the challenges that appeared during the project were not discussed in the publication.

Similarly to the German study [2], the benefits and challenges of international databases on AMU and AMR are discussed somewhat tentatively. Two exceptions are the publications of Ferreira et al. [6] and Geneviève et al. [8]. They address challenges by comparing databases of countries such as Switzerland and Denmark. The challenges were categorized into ethical-legal, technical, soft and other aspects. including common challenges in database management such as privacy, autonomy in federal systems, data privacy rules, data quality issues, other access rights, anonymization, informed consent and data linkages, among others (see further in [5]).

Another topic that is frequently discussed is the quality of the collected data. Lastein et al. [10] focus on the documentation of treatment data and indicate that there is more variability behind a digit than the number itself can express. Variability starts with a “cultural framework” of caring for livestock and its regional legal requirements, the veterinarian’s decision to administer treatment or not and, furthermore, which antimicrobial drug should be selected (see also [1,11]). Similarly, other scholars [1,2,6] discuss the quality of data as a matter of general or automated plausibility checks that can be implemented by techniques such as validation of formal requests, checking of invalid category combinations (e.g., antibiotics for diarrhea filed for skin problems) or identifying missing data. Sanders et al. [1] suggest standardizing available inputs as a step to ensure data quality. However, before standardization is possible, studies from Geneviève et al. [8] and other researchers [6,12,13] outline that good data collection requires open and collaborative communication to actually assess where reusability or interoperability is hampered and a shared view can be found.

With regard to data quality, another aspect that is discussed is how illiteracy influences the data collection process, impacting both the ability to read and understand the importance of reporting certain details about the process or antibiotics administered for database documentation [11,14]. Besides illiteracy, the study by Arnold et al. [11] points out that a lack of infrastructure may be one of the key challenges to consider when assessing AMU and AMR in rural areas. However, even if (technical) infrastructure exists, using these systems may pose several challenges to documenting AMU [8].

### 2.2. Presenting the VetCAb-ID Database and Functions

In a joint study by the University of Agriculture in Faisalabad (UAF) and the Department for Biometry, Epidemiology and Information Processing (IBEI), the feasibility of monitoring AMU using VetCAb-ID was assessed. This section begins with a description of the basic features of the database. In the next section, interview results regarding the documentation process, starting at the farm level, will be presented.

**Figure 1:**
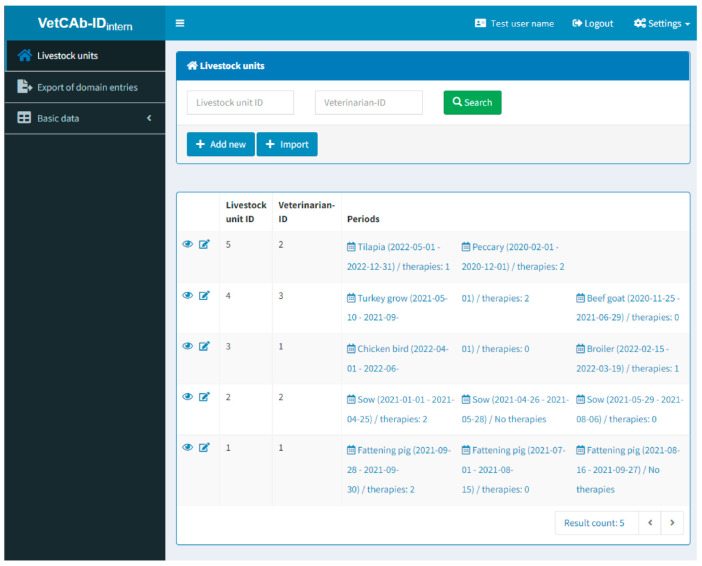
Overview of entries by test user in the VetCAb-ID database.

VetCAb-ID is a database used at a global level that aims at providing a web-based infrastructure to users in the field of AMU in the veterinary field (see more context information in the “Methods” section). As one of the first steps, the administrators of the IBEI set up a domain for users to enter and analyze data jointly. A domain can be a project at a national, regional or university level and may or may not allow other domains to view or use the data within that domain. The database offers a set of basic forms to file farms (and animals from these farms), harvesting periods and treatments for these animals. To file treatments, animals and farms, the database offers predefined items that can be modified if they are incomplete for a domain. For each user, the database provides an overview of filed farms, veterinarians supervising the farms, animals and therapies (treatments) filed during a certain period (see Figure 1).

Preparing the VetCAb-ID database for international users requires extensive translation and discussions about appropriate terminology and the extent to which data input is compulsory. Moreover, defining roles and distributing rights in the database for administrators, domain or data editors and readers of entries in the database is needed to establish data security issues.

To overcome the common challenges faced by new users, three paper-based manuals and online training videos have been created and shared to explain how to use the VetCAb-ID database. The manuals address project norms, rights, legacies and data structures. They provide a checklist for what to consider when setting up the database and which user roles may be distributed. Roles include, for example, administrators (who can view all data, delete data and set up projects and users), domain editors (who are project leaders and may edit information, periods and therapies of projects and farms), data editors (who are project members and may enter therapy data for farms) and readers (who are people allowed to view the data in the database).

**Figure 2:**
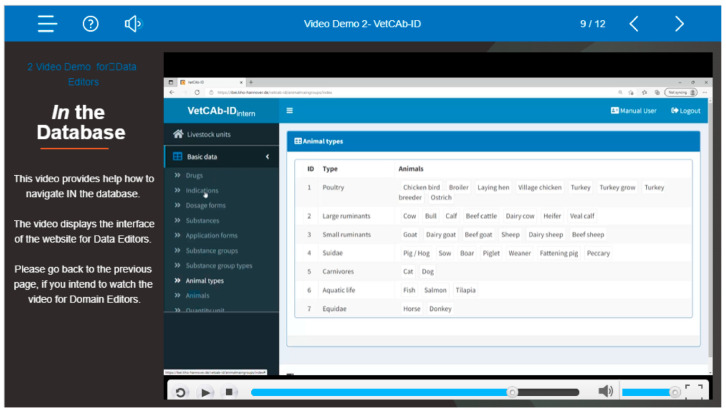
Screenshot from the second learning video for the VetCAb-ID database.

Besides written manuals, learning videos are available to explain and demonstrate how to use VetCAb-ID and how to analyze and export data from the platform [15]. Figure 2 provides a screenshot of a video demonstration from the second learning video for VetCAb-ID.

**Figure 3:**
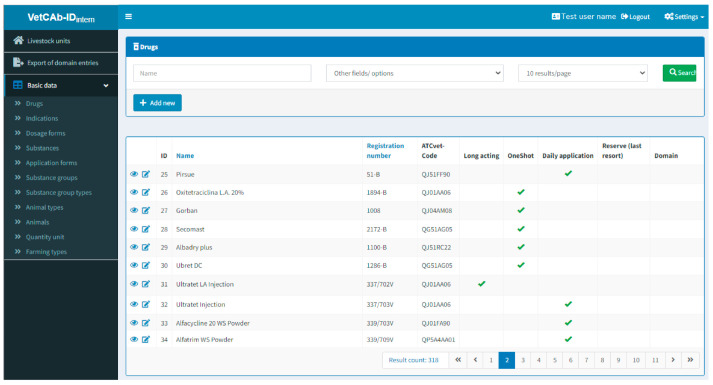
Drugs entered and available for several domains (anonymized). The dropdown menu “Basic data” is opened. For each of the options in the dropdown list, a set of predefined entries is provided to users.

Once users are familiarized with VetCab-ID, the database supports correct data input through the use of automated and manual quality checks. These checks comprise the provision of lists for entries of drugs, animals, dose units and indications, among other prefilled entry fields (harmonized input of data; see Figure 3).

**Figure 4:**
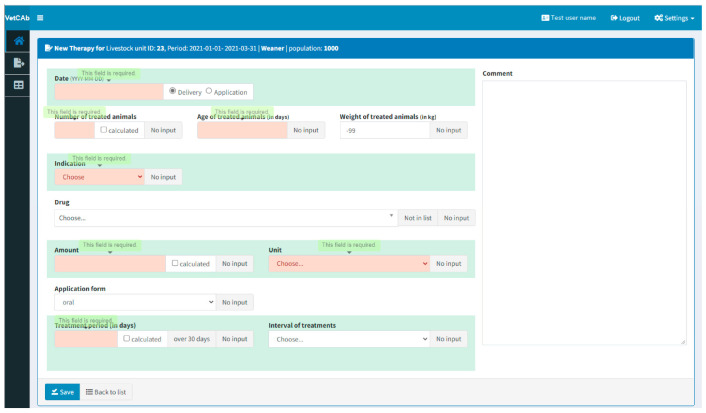
Form for entering therapies once livestock, period, kind of animal and population are registered. The picture shows an entry for a population of weaners from a test user.

In addition, consistency is checked with the help of calculations, such as the calculation of the correct periods for harvesting when entering a therapy (input consistency checks). In cases of false entries, the user is informed (see Figure 4) and may contact the IBEI to assess the problem if the inconsistency remains unclear or if the system needs to be adapted for new input combinations.

In summary, the main challenge during the development of VetCAb-ID for international users was to balance adaptive and predefined entry functionalities needed to harmonize data across domains on the one hand, while being responsive to domain/country-specific needs on the other. Furthermore, a balance between the administrative leadership of the IBEI (as the provider of the platform), the data sovereignty of the domain leaders and the distribution of rights across domains had to be found.

### 2.3. Results from the Expert Interview

In the joint study by the UAF and IBEI, AMU documentation begins with field veterinarians in Pakistan (poultry consultants), who first take notes and record data on-site at farms. The objective of documenting data on-site at farms is to overcome the lack of antimicrobials sales data, a common challenge in low- and middle-income countries (LMICs) [9]. The veterinarians use printed forms that list the required data items. These items correspond to the input fields of the VetCAb-ID database to ensure that a complete entry can be provided. To ensure data quality, field veterinarians were trained by the UAF in using the printed forms. Data collection forms for each flock of broilers are provided to the UAF team on a monthly basis.

The team at the UAF consists of several researchers. Two people enter the data from the forms into the VetCAb-ID database and the other people collect the information on antimicrobial product details from the retail market or through online searching. Since data can be entered into VetCAb-ID directly, the database is used as a project repository without the need for additional infrastructure or systems. The database allows for data to be exported in interchangeable formats so it can be further used in analysis systems.

Due to the challenges that appeared during the use of VetCAb-ID in Pakistan, administrative aspects need to be mentioned. One example is IBEI’s decision to keep the right to edit or remove incorrect entries to maintain database consistency. The UAF considered not having the right to edit and remove incorrect entries in the VetCab-ID platform a challenge. Small typos could not be corrected independently. Instead, an email needed to be sent to ask the administrator to solve the issue.

In general, the UAF indicated that additional, more predefined forms would be needed in order to standardize data monitoring in the field. One reason is that the required information for AMU/AMR is not readily available in Pakistan, and the more information that is filed at the farm level, the more complete the records that are filed online can be. A second reason is that a harmonized form would enable it to be shared across multiple projects if this approach were to be scaled on a national level. For example, duplications of entries (such as the type of antimicrobial drugs) could be avoided.

Concerning the documentation process, another challenge for the UAF was explaining to farmers (and veterinarians) why information such as the brand and the total amount of active ingredients in an antimicrobial drug needed to be recorded in detail. Explaining why recording is significant touches upon the common understanding of the importance of how AMU is protecting both local and global health. Related to this point, the UAF indicated that a commitment to report in a timely manner was a challenge:

*“For field veterinarians the collection of antimicrobial usage data on our given preforms was not their main priority. Since data collection was voluntary, sometimes we faced difficulties in getting data timely as it was not compulsion. Furthermore, the COVID-19 outbreak also affected the logistics and slowed the data collection and entry”*—Expert.

To overcome the aforementioned challenges, the UAF suggested shifting from paper-based data gathering to a digital application to report data directly. The system would create entries in the local languages and farmers would be able to create entries themselves. Real-time, direct reporting is considered an incentive, which leads to increased commitment:

*“It helps farmers to keep the farm records and to compare among different flocks and months”*—Expert.

To increase support, urging the Government of Pakistan to devise legislation and to direct farmers to record and report the AMU data was considered a step to overcome these challenges.

Regarding the VetCab-ID database, a simpler web-based database with a holistic approach (offering to file all types of indications for treatment and all kinds of animal species, for example) is the target. However, when asked about the benefits of using the platform, an expert summarized them as follows:

*“Earlier studies from our research group in Pakistan have studied antimicrobial usage data using treatment record and garbage-bins studies from food animals. Now our pilot VetCAb-ID project has provided us the first digital database of common antimicrobials brands, products and their indication in dairy and poultry farms in Pakistan. We are able to see descriptive results at the end of analysis.”*—Expert.

More information about entering and using specific data and their related benefits will be outlined in the next section.

### 2.4. Poultry Data of Pakistan in VetCAb-ID

For the UAF, using the VetCAb-ID platform is their first time using an international platform for recording data on AMU. As outlined in the previous section, using the database presented a challenge because the requested data were not readily available and needed to be generated first:

*“We added more than 146 antibiotics brands, composition, and dosage forms in the database.”*—Expert.

On the other hand, having this data filed offers benefits due to the resulting depth of information and results over time. Moreover, the antibiotics filed can be shared across domains (countries, projects), which reduces the need to generate new data. Further reported benefits relate to descriptive outputs such as:

*“The number of therapies, treatment frequencies (TF) and indications (diseases/prophylaxis). As researchers, this helped us in better understanding of collecting and reporting AMU data and the very details to consider in implementing AMU surveillance at farm-level or national level.”*—Expert.

In previous papers, AMU data were estimated on the basis of import/export data from the Pakistan Customs Authority [16]. As shown in Table 1, using the VetCAb-ID database allows for several additional details to be documented (only an excerpt of the filed information is provided. The data, including treatment frequencies, will be provided upon request).

Table 1 shows the date on which a specific type of drug was provided to animals, in what quantity and during which treatment period. Up to the specified date for a certain farm and period, the CAT is calculated and provided. As seen in the table, two farms used the same drugs during the same treatment period. The amount of drugs provided is also the same in both farms.

A beneficial feature of the database that can be observed in this respect is that entries (such as drug name, animal or indication of therapy) are linked automatically to a corresponding identification number (ID) in the VetCAb-ID platform. Since the ID lists can be accessed, modified and exported with the domain database sheets, this documentation eases later data processing and analysis (where text input such as indications would have to be converted into IDs manually). Moreover, IDs such as the drug name are linked to other information in the VetCAb-ID database (e.g., the ingredient ID in the table is linked to certain drug names). Hence, based on the drug name, the database allows the user to analyze related information that has not yet been manually entered from a single data point entry.

## 3. Discussion and Conclusions

Here, we present a case study of VetCAb-ID being used for documenting poultry data in Pakistan. Although Pakistan uses the database for more than one animal species, and despite the fact that VetCAb-ID is used by several countries, we chose to begin with this focused and qualitative case study in order to highlight the process of data entry and specific system features. To avoid the risk of presenting incomparable results, we conducted a screening literature review to align our results with the findings in other projects. In this regard, we found that our case study provides one of the first user reviews of an international database for AMU documentation. While previous publications have focused on comparing the functionalities between databases across different countries or methods, this study elaborates on the feasibility of monitoring starting in stables. Future studies should provide systematic literature reviews and multiple case studies to increase the generalizability of the findings. Specific topics related to the discussion of our results are addressed in the following section.

Regarding the documentation process, our study found that a paper-and-pen approach to recording data in stables is sufficient in Pakistan. Although commitment to submit related documents in a timely manner is an issue, data can be uploaded online regularly by the UAF team. In the future, current users in Pakistan aim to document AMU in stables with the help of digital systems. Since infrastructure may be a severe challenge to documentation as outlined in previous studies [11,17] and several other approaches to data collection exist (e.g., [14]), the goal to document data digitally on a farm level may not be generalized to a global level. However, other studies have outlined that if the internet connection is weak, a digital offline recording system that can be synchronized when connected to the internet at a later time may be a compromise [2]. Hence, the positive evaluation of a digital input system needs to be further investigated in the future, especially with regard to the question of how local and offline databases need to be organized if the number of stakeholders involved increases (e.g., [18]).

In relation to the digital input of primary data, another surprising result of this study is that VetCAb-ID seems to be one of the few global databases that allows users to collect, save, export and use their data independently (and, depending on the agreement of other users, other data as well; see the description of user roles in Section 2.2.). Since VetCAb-ID members’ experiences are positive, both the data sharing and the definition of user roles seem to be a solution to avoid generating more “data silos” [8]. However, VetCAb-ID does not have experience with data being provided by farmers directly. Redding et al. [14] pointed out that farmers’ self-reporting may be prone to bias. Moreover, studies in the literature review underline that documenting specific information, such as the reason for treatment or the illness level, might be needed for comparable data documentation as well [1,11]. Hence, whether new roles, responsibilities, data categories and checking processes are needed needs to be explored in future projects.

Another finding of this study is that the effort to provide the necessary input in VetCAb-ID is significant since some data may not be readily available for documentation. Despite this effort, the harmonized approach to filing therapies is evaluated positively. To be more precise, even more predefined categories, forms and processes are needed. A clear benefit that can be derived from this is that sharing entries among database users leads to greater collaboration between stakeholders. With a huge stock of predefined, readily available input categories, database users can focus on specifying the database to their own means (instead of basic entries). Hence, regarding the discussions in the literature on whether to extend the harmonization of data records [1,4,8], this study suggests that harmonization is needed and wanted so long as flexibility for specific program features is granted.

This case study does not provide a discussion on how AMR or One Health data can be integrated and documented in a joint approach (e.g., [19,20]). Furthermore, no detailed list of the properties (requirements and related decision processes) of the VetCAb-ID database is provided. Sanders et al. [1] and Mesa Varona et al. [3] provide respective publications and a wealth of suggested requirements for future (and improved) databases to come. Hence, another core finding from this case study is that it is time to come together and formulate a joint view regarding the requirement of documentation for AMU/AMR on a global level. By collating the challenges and benefits of certain functionalities, a best practice catalog for AMU and AMR databases on an international level could be defined, which would help future projects to avoid pitfalls and provide an edge for antimicrobial stewardship.

## 4. Materials and Methods

### 4.1. General Framework and Study Targets

This publication is embedded in the project “Veterinary Consumption of Antibiotics-International Documentation” (VetCAb-ID). The project runs under the umbrella of the WHO Collaboration Center for research and training on health at the Human–Animal–Environment Interface (WHO CC HAEI) at the IBEI of the University of Veterinary Medicine Hannover (TiHo). The long-term goal is to establish a concept for a One Health approach and to report AMU and AMR data jointly online. The database (VetCAb-ID) for this project is hosted by the IBEI. It was built in response to a sentinel approach of collecting antimicrobial usage data in German farms [2]. Currently, VetCAb-ID enrolls users from institutes in Pakistan, Zambia, Chile and Saint Kitts/Nevis.

The data for this publication stems from a joint feasibility study of the UAF in Pakistan and Germany. The key research question is: What can we learn from using an internationally shared database for documenting the use of antimicrobial drugs across countries? The study design to answer this question is a mixed-method case study.

A case study is a systematic compilation of entities bound, for example, by a project [21,22]. In this study, mixed methods refer to qualitative (expert interviews, screening literature review) and quantitative data (descriptive data on poultry flocks). The reason for the inclusion of multiple data sources is that qualitative research based on one source, such as primary data obtained from interviews only, may be prone to bias. Summarizing, comparing and triangulating the findings from these sources is one way to ensure the quality of the research results [22,23].

### 4.2. The Case Study Context

The UAF in Pakistan has been one of the active partners using VetCAb-ID since the end of 2020. In LMICs, documenting the use of antimicrobial agents is rare. Pakistan has expanded its activities to address the overuse of antibiotics, especially since a significant part of the WHO-classified high-priority critically important antimicrobial (HPCIA) agents in human medicine are used in farm animals. In 2017, a National Action Plan (NAP, [24]) was launched to improve awareness, strengthen the knowledge on AMR/AMU in the country, reduce the incidence of infection and optimize the use of antimicrobials in human and animal health [24]. Recent publications highlight the need for stronger regulation and enforcement due to the high overuse of antibiotics, particularly in chicken [9,16]. Since the NAP has not yet launched a shared dashboard for data sharing, the UAF joined the VetCAb-ID project to collect its project data.

### 4.3. Collecting and Analyzing Qualitative Data

To consider the latest publications on the topic, a screening literature review was performed following Webster and Watson [25]. Keywords for the first search through the VetSearch platform (a bibliographic database at the TiHo) were: veterinary* AND antibiotic* AND antimicrobial* AND record* AND monitor AND collect* data AND feasibility AND database, resulting in 165 records with no relevant output. Subsequently, the search was tailored and terms including case study, data or medical records, data entry or collection were included to gather a broader output. The output was controlled with a complementary search through Google Scholar to expand the resources. Eventually, the results with case studies were included if they were available and addressed the topic (use of databases and documentation processes, in particular). The analysis aimed to compare, concept-wise, how publications help to answer the research questions of the study [25].

To provide an adequate account of the experiences with the VetCAb-ID database in particular, the UAF team acted as experts in an interview [26]. A brief questionnaire was compiled to assess the process, strengths and weaknesses and generate a reflection on experiences with the database. The answers were provided in writing and open questions were discussed in person via online meeting platforms. The interview was used to triangulate and complement the findings in the literature, as presented in the “Results” section.

### 4.4. Collecting and Analyzing Quantitative Data

To illustrate the functionalities of VetCAb-ID, extracts from the database and a descriptive analysis of the data in VetCAb-ID for poultry farms in Pakistan were generated. The study population stems from two large-scale commercial broiler farms with more than 25,000 chicken birds. The farms are providing AMU data on a voluntary basis. In total, two or three poultry production passages at each farm were documented. The type of poultry is defined as chickens (age: 0–42 days) raised for meat production. While the information about the farm, species and periods is filed in advance by the UAF, the data about therapies are filed by veterinarians, provided to the UAF, entered by UAF members in the database and cross-checked by IBEI members. The main calculations focus on the average treatment frequency (TF). The TF is calculated by adding up the number of recorded therapies, considering the active substance in drugs, and dividing by the population size [27]:

TF = ∑ number of treated animals × treatment days in period/population size(1)

This definition (1) is in line with treatment incidence, but it assumes UDD instead of DDD and body weight under therapy instead of average body weight [27].

## Figures and Tables

**Table 1:** Brief output for farm_id 84, therapy ID: 87, 89, (broiler, population of 28,000 chickens) and farm_id 95, therapy ID: 412, 413 (broiler, population of 25,000 chickens).

Date	Drug_Id	Drug_Name	Ingredient_Id	Amount	Quantity Unit_Id	Treatment Period	CAT ^1^
1 June 2021	381	Enrotil Oral Solution	1804	3000	mL	3	84,000
16 June 2021	382	Kepro Tylo-Dox Extra W/S	2316	3000	g	4	112,000
18 October 2021	381	Enrotil Oral solution	1804	3000	mL	3	75,000
21 November 2021	382	Kepro Tylo-Dox Extra W/S	2316	3000	g	4	100,000

^1^ CAT: cumulative number of animals treated times treatment days.

## Data Availability

Since this is a feasibility study, data on poultry need to be analyzed with caution. To ensure this, the full dataset from this study will be made available upon request.
